# Unintentional injection to the bone with a pediatric epinephrine auto-injector

**DOI:** 10.1186/s13223-018-0257-6

**Published:** 2018-08-20

**Authors:** Mariam Ibrahim, Harold Kim

**Affiliations:** 1 Department of Engineering, University of Guelph, Guelph, ON Canada; 2 Department of Medicine, Division of Clinical Immunology & Allergy, Western University, 525 Belmont Ave W, Ste 205, Kitchener, ON N2M 5E2 Canada

**Keywords:** Anaphylaxis, Children, Skin-to-bone distance, Epinephrine auto-injector, Bone injection, Needle length

## Abstract

**Background:**

Skin-to-bone distance (STBD) in children prescribed a pediatric epinephrine auto-injector (EAI) for anaphylaxis is not commonly measured in practice. Recent evidence suggests that children with STBD less than the exposed needle length of available pediatric EAIs (dose: 0.15 mg, needle length: 12.7 mm) are at risk for unintentional injections to the bone during their use for an allergic emergency.

**Case presentation:**

Described here is a case of a female child with multiple food allergies prescribed a pediatric EAI (0.15 mg EpiPen Jr^®^) who experienced an unintentional injection to her femur. The patient’s STBD at the recommended injection site (vastus lateralis) was shorter than the needle length of her prescribed EAI (12.7 mm) at the time of the injury (age: 7, height: 122 cm; weight: 25 kg), even though her weight was within the indication for this EAI (15–30 kg). The patient and her family were made aware of the risk of unintentional bone injection at the time the EAI was prescribed.

**Conclusions:**

Some children, even those at an appropriate weight per the indication of available pediatric EAIs (0.15 mg), may be at risk for unintentional injections to the bone. The effects of an unintentional bone injection with an EAI can have lasting effects on a child, including pain. Healthcare providers who prescribe pediatric EAIs for any child should consider evaluating this risk, inform patients and parents of the risk, and take measures to potentially mitigate unintentional bone injections. For some children, an EAI with a shorter needle length may be a more appropriate choice of treatment for anaphylaxis.

## Background

EAI-related injuries in children receiving epinephrine during an allergic emergency are poorly studied, and reports to that effect are sparse. Although many of the unintentional injuries that occur with EAIs are at the expense of the person performing the injection while self-administering, administering to another person, familiarizing themselves with the device, disposing the device, or during training sessions [1, 2], injuries to pediatric patients do occur but are rare. In a report published in 2016, Brown et al. describe 22 EAI-related injuries in children (age range 1–11 years; weight range 7–48 kg). While the majority of these injuries involved lacerations to the thigh while using the EpiPen Jr^®^ (i.e., leg flailing during the injection), two patients experienced stuck needles after administration; one in the lateral thigh of a 5-year old patient, and one in the tibia of an 8-year old patient [3]. This study alone prompted changes to all pen-style EAI package inserts to include verbiage on the need to restrain a child’s legs during an injection.

The appropriate exposed needle length for a pediatric EAI has become an issue of recent controversy [4, 5]. In children < 15 kg, recent evidence has shown that the current needle length of pediatric EAIs (dose: 0.15 mg; needle length: 12.7 mm; indication: 15–30 kg) may put children at risk for unintentional bone injections [6, 7]. These evaluations are based on skin-to-bone distance (STBD) measurements by sonography at the recommended injection site (vastus lateralis) in children < 15 kg. One study demonstrated that 29% of children < 15 kg (N = 100) had an STBD < 12.7 mm, which would put them at risk for an unintentional bone injection [7]. In a subgroup of children < 10 kg in this study, 60% (n = 25) would be at risk for unintentional injection to the bone based on their STBD. A more recent study showed that 43.1% of patients (≥ 7.5–15 kg; N = 51) would be at risk of an unintentional bone injection if using one of the available pediatric EAIs [6]. This study determined that the appropriate needle length for children weighing ≥ 7.5–15 kg would be 7–8 mm. In November 2017, the U.S. Food and Drug Administration approved an EAI with a needle length of 7.27 mm for use in children ≥ 7.5–15 kg [8, 9]. However, it is likely that some children between 15 and 30 kg may have an STBD less than the needle length of available pediatric EAIs (12.7 mm) indicated for this weight range. A case of unintentional injection to the bone involving a female patient with an STBD less than the needle length of her pediatric EAI at the time of use is presented.

## Case presentation

MS is a 10-year-old female patient diagnosed with multiple food allergies and anaphylaxis (Table 1). MS has a family history of atopy (both parents). MS’s index allergic event was at 9 months of age when she presented with urticaria after contacting milk and cheese. By the age of 5, MS had been diagnosed with milk, peanut, and tree nut allergies based on clinical history, skin prick testing, serum-specific IgE testing, and oral food challenge (OFC). Also, she has dust mite and pet allergies.

**Table 1:** Notable case findings related to food allergy testing

Age, years	≤ 5	6	9
Food allergy testing	SPTs positive to baked milk (6 mm) SPTs positive to real pistachio and to extracts of cashew (6 mm) SPTs positive to dust mites, molds, cat, dog SPTs to peanut and almond were borderline SPTs to soy, shrimp, and wheat were negative OFC to peanut positive. Rash appeared on face swollen conjunctiva after 8 peanuts but then resolved	Serum-specific IgE to milk was positive at 27.40 Serum-specific IgE to cashew was positive at 10.80 This would suggest a risk of about 95–100% for anaphylaxis with a significant ingestion of either allergen	Serum-specific IgE to milk was 17.30 Serum-specific IgE to cashew was positive at 14.40 Serum specific IgE to pistachio was positive at 18.10 This would suggest a risk of about 95–100% for anaphylaxis with a significant ingestion of these allergens

Since early in life, she was prescribed a pediatric EAI (EpiPen Jr^®^; 0.15 mg) in case of an allergic emergency. The first use of her prescribed EAI for MS was at age 6, and she has had 2 subsequent allergic emergencies requiring the use of her EAI as of the date of this report (Table 2). At 6 years old, her height was 117 cm, weight was 17.7 kg, BMI was 12.9, and her STBD was 10.7 mm by ultrasound of the right mid-anterolateral thigh. At 7 years old, around her second event requiring the EpiPen Jr^®^, her height was 122 cm, weight was 25 kg, and her STBD was not recorded. After this event, and during her observation period in the hospital, MS complained of pain immediately in her right thigh at the injection site. X-ray and ultrasound results were negative. It is believed that MS suffered an unintentional bone injection with her EAI based on clinical presentation following the allergic emergency. At 9 years old, her height was 141 cm, weight was 30 kg, and her STBD was 12.1 mm. In 2017, MS was evaluated again for pain and discomfort in her right thigh.

**Table 2:** Notable case history related to pediatric EAI use

Age at event, years	6 years (2013)	7 years (2014)	9 years (2017)
Height (cm)	117	122	141
Weight (kg)	17.7	25	30
STBD (with compression, mm)	10.7	11.1	12.1
Suspected/known allergen	Milk	Cake with cashew	“Snack Pack” bakery shop dessert (tapioca, carrageenan)
Symptoms/acute care	MS ingested milk and her caregiver administered the EpiPen Jr^®^ because she thought MS complained of throat itchiness	MS had immediate shortness of breath, hives, throat tightness, and lip swelling Her mother gave her the EpiPen Jr^®^ in the right mid-anterolateral thigh Epinephrine worked immediately, and she improved within 30 s MS had transient shakes, tachycardia, fatigue lasting approximately 3 min MS was taken to and observed in the Emergency Department. MS complained of pain immediately in her right thigh at the injection site, which has persisted over time X-ray and ultrasound were negative	MS had an immediate throat swelling and shortness of breath MS was given 2 doses of epinephrine Her symptoms improved within minutes
Follow up case notes	In August 2017, MS was examined for bony lesions, fracture, or soft tissue disorders that could explain her continued discomfort and pain The previous allergic emergency in 2014 requiring the use of her EAI was noted Negative X-ray and ultrasound of right femur and no evidence of muscular injury or hematoma. No discrete solid or cystic lesions It was noted that she was skeletally immature		
Long-term management	Keep prescribed EpiPen Jr^®^ available Instructions on how to give the EpiPen Jr^®^ without full muscle compression Continue strict avoidance of milk, raw peanut, cashew, hazelnut, pecan, and pistachio		

The indication for the EpiPen Jr^®^ is for children between 15 and 30 kg and this device has a needle length of 12.7 mm. MS and her parents were made aware that use of her prescribed EAI could cause an unintentional injection to the bone using the standard injection technique with compression. It was suggested that, prior to injection, the vastus lateralis muscle be squeezed as to avoid full compression while using her EAI because of the high risk of unintentional bone injection.

This case of possible bone injection was reported to Health Canada.

## Conclusions

Needle length of currently available EAIs remains an open area of inquiry and investigation [4, 5]. Unfortunately, there is little evidence to support age-, weight-, thigh circumference-, or BMI-specific needle length recommendations since the suitability of these biomarkers for this purpose has not been thoroughly investigated in large studies. This remains an area of unmet need. Previous work has shown that the risk of overpenetration with needles that are 25 mm and 31.75 mm in length in children ≥ 1 year for routine vaccinations is 11 and 39%, respectively [10]. Currently, the CDC recommends vaccination to the vastus lateralis at a 90-degree angle with a 15.8 mm needle for infants up to 12 months and a 25.4 mm needle for children 1 month and older, and either a 15.8 mm (deltoid) or 25.4 mm needle (thigh) for children 1–2 years old [11]. With manual delivery by needle and syringe, the angle of entry can be altered to correct for short STBD if suspected. However, pediatric EAIs have been engineered to be used with compression, as instructed by their prescribing information. Even with an exposed needle length of 12.7 mm, the needle may be too long for many children as more recent evidence has shown [6, 7].

In this case report, the subject’s parents and allergist believe that the EAI delivered at age 7 years hit and possibly penetrated the bone. Although, we could not confirm this with radiographic evidence, there was strong clinical evidence to support bone injection. First, bone pain and tenderness at the injection site was present immediately after the injection lasting for at least 2 years. Also, she had possible epinephrine side effects after the EAI injection at age 7. She had shakes, tachycardia and fatigue immediately after her allergic reaction and epinephrine delivery. The symptoms could have been due to the anaphylaxis or due to side effects of intramuscular delivery of epinephrine. We believe that the epinephrine may have been delivered through the cortical bone into the intraosseous space, which could lead to immediate intravenous delivery of the drug. At the time of the unintentional bone injection (patient height: 122 cm; patient weight: 25 kg), the patient’s STBD with compression was less than the needle length (12.7 mm) of her prescribed pediatric EAI (EpiPen Jr^®^ 0.15 mg). In this case, we had previously measured STBD and knew that there was a risk for unintentional bone injection in this patient.

In conclusion, our case illustrates that a risk for unintentional bone injection of epinephrine with currently available EAIs exists for children between 15 and 30 kg. However, most healthcare providers do not perform ultrasound studies on children to determine STBD. Therefore, they are unlikely to be aware of the risk for unintentional bone injections with currently available pediatric EAIs; i.e. devices indicated for children 15–30 kg. Unintentional injection of epinephrine to the bone in this case may have caused immediate systemic side effects as well as symptoms of pain and discomfort that persisted in the patient well after the initial adverse event. EAIs should be designed taking this risk into consideration.
